# Small-fiber polyneuropathy (SFPN), a common underlying diagnosis in syndromes involving unexplained chronic pain and multi-system symptoms

**DOI:** 10.1186/1744-8069-10-S1-O12

**Published:** 2014-12-15

**Authors:** Anne Louise Oaklander, Heather Downs, Zeva Daniela Herzog, Max Klein

**Affiliations:** 1 Department of Neurology, Massachusetts General Hospital, Harvard Medical School, Boston, MA 02114, USA; 2 Departments of Pathology (Neuropathology), Massachusetts General Hospital, Boston, MA 02114, USA

## Background

Syndromes involving unexplained chronic widespread pain (CWP) and multi-system symptoms are common, with 1-5% prevalence for fibromyalgia alone. They more often affect females and cause disability and high costs [1-3]. Other common syndromes include chronic fatigue, seronegative Lyme, and Gulf War Illness. Fragmentary syndromes include TMJD, POTS, CRPS, irritable bowel). These syndromes are particularly devastating in children and young adults, where they interfere with education and development and disrupt entire families [4-6]. SFPN is known to cause CWP and multi-system complaints in older adults. Unlike the syndromes above, SFPN can be objectively diagnosed by measuring innervation in lower-leg skin biopsies, and autonomic functions testing (AFT) of heart rate, blood pressure and sweating [7]. SFPN has several established causes including diabetes, infections, cancer, and toxins. Many causes are diagnosable, treatable, and sometimes curable [8]. Our work suggests that unrecognized SFPN contributes to several syndromes involving CWP and multi-organ symptoms.

## Materials and methods

With IRB permission, we retrospective analyzed the medical records of 41 patients with onset of unexplained CWP and multisymptoms before age 21; most had objective testing for SFPN [9]. We also prospectively studied 27 adult patients with fibromyalgia and 30 healthy volunteers using history, examination, skin biopsies and AFT [10].

## Results

Retrospective chart review identified definite (in 59%) and probable SFPN (in 17%) among the young patients with onset before age 21 [9]. We characterized the clinical features, diagnostic, and treatment options for this new early-onset SFPN. Studying children, who lacked the typical causes of late-onset SFPN, implicated autoimmune causality in most. Among patients treated with immunomodulatory therapies, pain and other symptoms improved in 2/3 [9]. Among adults with fibromyalgia, 41% of skin biopsies from subjects with fibromyalgia vs. 3% of biopsies from controls were diagnostic for SFPN, and symptom and examination scores were higher in fibromyalgia subjects than in controls (all P ≤ 0.001) [10]. All fibromyalgia patients diagnosed with SFPN then had blood tests for all known causes [8]. None had diabetes but 62% had test-results consistent with dysimmunity, and some had genetic causes [10]. Other laboratories have now also linked fibromyalgia to SFPN [11-15].

## Conclusions

Some patients with unexplained widespread pain and multi-system syndromes such as fibromyalgia have objectively diagnosable SFPN. SFPN can affect children and young adults, not just older adults. Multiple lines of evidence suggest that early-onset SFPN has novel causes that can be treated. The prevalence of SFPN among TMJD patients is unstudied.

## Disclosures

None of the authors have any conflicts of interest.

## References

[B1] Lindell L Bergman S Petersson IF Jacobsson LT Herrstrom P Prevalence of fibromyalgia and chronic widespread pain Scand J Prim Health Care 2000 18 149 153 10.1080/028134300453340 11097099

[B2] White KP Speechley M Harth M Ostbye T The London Fibromyalgia Epidemiology Study: the prevalence of fibromyalgia syndrome in London, Ontario J Rheumatol 1999 26 1570 1576 10405947

[B3] White KP Speechley M Harth M Ostbye T The London Fibromyalgia Epidemiology Study: direct health care costs of fibromyalgia syndrome in London, Canada J Rheumatol 1999 26 885 889 10229411

[B4] Buskila D Pediatric fibromyalgia Rheumatic Disease Clinics of North America 2009 35 253 261 10.1016/j.rdc.2009.06.001 19647140

[B5] van Geelen SM Bakker RJ Kuis W van de Putte EM Adolescent chronic fatigue syndrome: A follow-up study Arch Pediatr Adolesc Med 2010 164 810 814 20819962 10.1001/archpediatrics.2010.145

[B6] Mikkelsson M El-Metwally A Kautiainen H Auvinen A Macfarlane GJ Salminen JJ Onset, prognosis and risk factors for widespread pain in schoolchildren: A prospective 4-year follow-up study Pain 2008 138 681 687 10.1016/j.pain.2008.06.005 18701216

[B7] England JD Gronseth GS Franklin G Carter GT Kinsella LJ Cohen JA Practice Parameter: Evaluation of distal symmetric polyneuropathy: role of autonomic testing, nerve biopsy, and skin biopsy (an evidence-based review). Report of the American Academy of Neurology, American Association of Neuromuscular and Electrodiagnostic Medicine, and American Academy of Physical Medicine and Rehabilitation Neurology 2009 72 177 184 10.1212/01.wnl.0000336345.70511.0f 19056667

[B8] England JD Gronseth GS Franklin G Carter GT Kinsella LJ Cohen JA Practice Parameter: Evaluation of distal symmetric polyneuropathy: role of laboratory and genetic testing (an evidence-based review). Report of the AAN, AANEM, and AAPMR Neurology 2009 72 185 192 10.1212/01.wnl.0000336370.51010.a1 19056666

[B9] Oaklander AL Klein MM Evidence of small-fiber polyneuropathy in unexplained, juvenile-onset, widespread pain syndromes Pediatrics 2013 131 e1091 e1100 10.1542/peds.2012-2597 23478869 PMC4074641

[B10] Oaklander AL Herzog ZD Downs HM Klein MM Objective evidence that small-fiber polyneuropathy underlies some illnesses currently labeled as fibromyalgia Pain 2013 23748113 10.1016/j.pain.2013.06.001 PMC3845002

[B11] Üçeyler N Zeller D Kahn AK Kewenig S Kittel-Schneider S Schmid A Small fibre pathology in patients with fibromyalgia syndrome Brain 2013 23474848 10.1093/brain/awt053

[B12] Albrecht PJ Hou Q Argoff CE Storey JR Wymer JP Rice FL Excessive peptidergic sensory innervation of cutaneous arteriole-venule shunts (AVS) in the palmar glabrous skin of fibromyalgia patients: Implications for widespread deep tissue pain and fatigue Pain Med 2013 14 895 915 10.1111/pme.12139 23691965

[B13] Serra J Collado A Sola R Antonelli F Torres X Salgueiro M Hyperexcitable C nociceptors in fibromyalgia Ann Neurol 2013 24243538 10.1002/ana.24065

[B14] Giannoccaro MP Donadio V Incensi A Avoni P Liguori R Small nerve fiber involvement in patients referred for fibromyalgia Muscle Nerve 2013 24469976 10.1002/mus.24156

[B15] Caro XJ Winter EF Evidence of abnormal epidermal nerve fiber density in fibromyalgia: Clinical and immunologic implications Arthritis Rheumatol 2014 24719395 10.1002/art.38662

